# Control of stochastic and inverse stochastic resonances in a liquid-crystal electroconvection system using amplitude and phase noises

**DOI:** 10.1038/s41598-023-44043-4

**Published:** 2023-10-06

**Authors:** Jong-Hoon Huh, Masato Shiomi, Naoto Miyagawa

**Affiliations:** Department of Physics and Information Technology, Faculty of Computer Science and Systems Engineering, Kyushu Institute of Technology, Fukuoka, 820-8502 Japan

**Keywords:** Physics, Condensed-matter physics, Statistical physics, thermodynamics and nonlinear dynamics

## Abstract

Stochastic and inverse stochastic resonances are counterintuitive phenomena, where noise plays a pivotal role in the dynamics of various biological and engineering systems. Even though these resonances have been identified in various systems, a transition between them has never been observed before. The present study demonstrates the presence of both resonances in a liquid crystal electroconvection system using combined amplitude and phase noises, which correspond to colored noises with appropriate cutoff frequencies (i.e., finite correlation times). We established the emergence of both resonances and their transition through systematic control of the electroconvection threshold voltage using these two noise sources. Our numerical simulations were experimentally confirmed and revealed how the output performance of the system could be controlled by combining the intensity and cutoff frequency of the two noises. Furthermore, we suggested the crucial contribution of a usually overlooked additional phase noise to the advancements in various noise-related fields.

## Introduction

Stochastic resonance (SR) is an attractive counterintuitive phenomenon induced by noise combined with a deterministic signal^1^, which enhances the output performance of unknown weak signals below the threshold of detection tools. Usually, SR shows a maximal output performance peak corresponding to the signal-to-noise ratio at a moderate optimal noise level. Since Benzi et al*.* first suggested this phenomenon and its underlying mechanism in a study on ice-age cycles^2,3^, SR has been extensively investigated in various fields, including physics^4,5^, chemistry^6,7^, biology^8,9^, information technology^10,11^, and brain science^12,13^. By contrast, an opposing phenomenon known as the inverse SR (ISR) was initially discovered in a neural system^14–16^, showing a minimal output performance peak at a moderate optimal noise intensity. Later, ISR was also reported in other systems such as ecological systems and during electroconvection (EC)^17,18^.

The mechanisms of both SR^1^ and ISR^19^ are generally described using an expanded Langevin equation as follows:

1 $$\frac{dx}{{dt}} = - \frac{{\partial [\phi (x) - A_{0} x\cos \Omega t]}}{\partial x} + \zeta (t).$$

In standard SR, the presence of a weak deterministic signal (i.e., *A*_0_ ≠ 0) and noise *ζ*(*t*) dictates that the potential function *ϕ*(*x*) for the system of interest must be bistable (i.e., two stable minima)^1^. Conversely, ISR can be obtained in the absence of a weak deterministic signal (i.e., *A*_0_ = 0) if the two minima have different depths and widths. Consequently, the reflection symmetry (*x* →  − *x*) in the quartic double-well potential is broken for ISR^19^, whereas the deterministic signal (*A*_0_ ≠ 0) breaks the symmetry in SR. In other words, the symmetry-broken potential is periodic (Ω ≠ 0) for SR and stationary (Ω = 0) for ISR^19^. In contrast to the standard SR introduced by Benzi et al*.,* Sutera suggested a pure noise-induced transition (*A*_0_ = 0) to explain the ice-age cycles^20^. Interestingly, nonstandard SR was also observed^19^ in the absence of a signal (*A*_0_ = 0), which is often referred to as coherent resonance^1^. Breaking the reflection symmetry of the two-minimum potential is essential to generate standard SR and ISR. Moreover, the noise *ζ*(*t*) in Eq. (1) is *additive*, i.e., independent of the variable *x*^21,22^. Similarly, *multiplicative* noise *ξ*(*t*) [i.e., *xξ*(*t*)] can provide SR^22–24^ and ISR^17^.

To the best of our knowledge, SR and ISR have never been observed concurrently in any actual system. To expand the use of SR and ISR into advanced applications, it is necessary to control both resonances and provide a transition between them, enabling the subsequent control of both desired and undesired system performances according to actual needs. In our previous studies, we confirmed the presence of both SR^25^ and ISR^17^ independently in a nonequilibrium system using different noises, i.e., *phase* noise for SR and *amplitude* noise for ISR. In the present study, we appropriately combined these two types of noise in order to control both resonances and the transition between them. In addition to the commonly used amplitude noise, our method utilized the usually overlooked phase noise^25,26^. Moreover, we used a *colored* noise with a finite autocorrelation time (*τ*_c_ ≠ 0)^17,21,22,27–31^, instead of the conventional quasi-white noise (*τ*_c_ ≈ 0)^2–13,27^.

In this report we demonstrate the transition between SR and ISR via a smooth variation of the output performance using ac-driven electroconvection in a nematic liquid crystal (NLC)^32–36^. Our findings show how to control monotonic and nonmonotonic variations of the EC threshold using colored amplitude and phase noises. The unusual nonmonotonic behavior of the threshold confirms the presence of both SR and ISR, providing maximal and minimal peaks of the EC pattern performance by efficiently facilitating and suppressing EC around a moderate optimal level of the amplitude and phase noises.

In this numerical study, the threshold voltage *V*_c_ of the EC was calculated using the one-dimensional Carr − Helfrich equations as follows^32–35^:

2 $$\dot{q} + \frac{q}{\tau } + \sigma_{{\text{H}}} \frac{V(t)}{d}\psi = 0,$$

3 $$\dot{\psi } + \lambda \left[ {E_{0}^{2} + \left( {\frac{V(t)}{d}} \right)^{2} } \right]\psi + \frac{q}{\eta }\frac{V(t)}{d} = 0.$$

**Figure 1:**
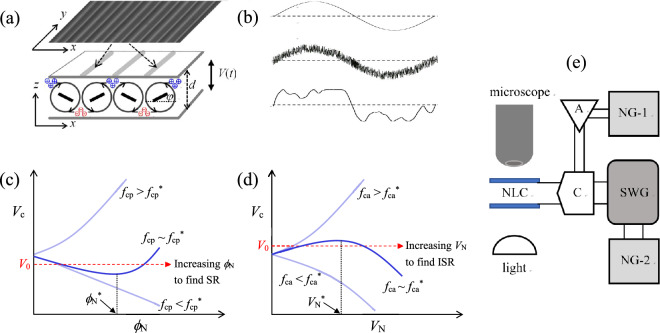
Colored noises-induced threshold variation of ac-driven electroconvection (EC). (**a**) A schematic representation of EC driven by Coulomb forces on electric charges (blue circle with plus sign, red circle with minus sign) in a nematic liquid crystal (NLC). The rods in the vortices of EC indicate the director ***n*** of the NLC. Above a threshold *V*_c_, EC is observed as a roll pattern (i.e., Williams domain) in the *xy* plane, which results from the periodic director angle *φ*(*x*). (**b**) Original sinusoidal voltage *V*(*t*) (i.e., *ϕ*_N_ = *V*_N_ = 0) (top) and voltages superposed by amplitude noise (i.e., *ϕ*_N_ = 0, *V*_N_ ≠ 0) (middle) and phase noise (i.e., *ϕ*_N_ ≠ 0, *V*_N_ = 0) (bottom). (**c**) Threshold voltage *V*_c_ as a function of phase-noise intensity *ϕ*_N_. (**d**) Threshold voltage *V*_c_ as a function of amplitude-noise intensity *V*_N_. (**e**) Experimental system for EC under two noise sources (*NG-1* noise generator for amplitude noise, *NG-2* noise generator for phase noise, *SWG* sinusoidal wave generator, *A* amplifier, *C* combiner). The functions of *V*_c_(*ϕ*_N_) and *V*_c_(*V*_N_) highly depend on the cutoff frequency *f*_c_ of the colored noise. In (**c**), nonmonotonic *V*_c_(*ϕ*_N_) is observed for *f*_cp_ ~ *f*_cp_^*^ of phase noise, indicating stochastic resonance (SR). In (**d**), reversed nonmonotonic *V*_c_(*V*_N_) is observed for *f*_ca_ ~ *f*_ca_^*^ of amplitude noise, indicating inverse stochastic resonance (ISR). See Refs^17,25^. for our previous results [*V*_c_(*V*_N_) and *V*_c_(*ϕ*_N_)] and corresponding EC pattern changes.

Here, *q*(*t*) and *ψ*(*t*) represent the space-charge density and curvature (*ψ* = *∂φ*/*∂x*) of the director for the deviation angle *φ* from the initial director ***n***_0_ (//$\hat{\user2{x}}$) at *V* = 0, respectively (Fig. 1a), and ***n*** corresponds to a unit vector indicating a locally averaged direction of the rod-like molecules of NLCs. The values of *τ*, *σ*_H_, *λ*, *E*_0_^2^, and *η* are determined by material parameters such as the electric and viscoelastic properties of the NLC with a thickness *d*. To examine the behavior of *V*_c_ in the presence of noise, we used an electric voltage *V*(*t*) with amplitude and phase noises, as shown in Eq. (4):

4 $$V(t) = \sqrt 2 V\cos \left[ {2\pi f_{0} t + \phi_{{\text{N}}} \xi_{{\text{p}}} (t)} \right] + A_{{\text{N}}} \xi_{{\text{a}}} (t).$$

Here, $\xi_{{\text{p}}} (t)$ and $\xi_{{\text{a}}} (t)$ correspond to phase and amplitude Gaussian-colored noises with cutoff frequencies *f*_c_ = 1/(2π*τ*_c_), respectively, and *ϕ*_N_ and *V*_N_ $= \sqrt { < (A_{{\text{N}}} \xi_{{\text{a}}} (t))^{2} > }$ are the amplitude and phase noise intensities, respectively. Technically, *f*_c_ can be controlled by low pass filters^25,27^. Hereafter, *f*_cp_ and *f*_ca_ indicate the cutoff frequencies for the phase and amplitude noises, respectively. In the EC system, the two noises superimposed on an initial ac field (Fig. 1b) is multiplicative^17,22,24,37^ because *V*(*t*) is directly coupled with the angle *φ* [in Fig. 1a for *ψ* = *∂φ*/*∂x* in Eqs. (2) and (3)]. The EC arises from *φ* = 0 (for *V* < *V*_c_) to *φ* ≠ 0 (for *V* > *V*_c_). Here, the additive noise *ζ*(*t*) in Eq. (1), corresponding to the thermal fluctuations in the NLC, can be neglected because it affects considerably less EC than the multiplicative noise^36,38^.

To better understand the present results, the *V*_c_ variations obtained using a single noise intensity (i.e., *ϕ*_N_ or *V*_N_) are schematically presented in Fig. 1c, d, as reported in our previous results^17,25^. Figure 1c shows that a colored phase noise with *ϕ*_N_ (*f*_cp_ ~ *f*_cp_^*^) at a fixed initial signal *V*_0_ (with a fixed *f*_0_) triggers the nonmonotonic behavior of *V*_c_(*ϕ*_N_), indicating SR^25^. The characteristic cutoff frequencies *f*_cp_^*^ and *f*_ca_^*^ are highly dependent on *f*_0_ (e.g., *f*_cp_^*^ < 2*f*_0_ and *f*_ca_^*^ < 20*f*_0_ for a typical NLC; *p*-methoxybenzylidene-*p*'-*n*-butylaniline (MBBA) was used in this study), and their applicable frequency range is *f*_c_^*^ ± Δ*f*_c_ (e.g., Δ*f*_cp_ =  ± 0.5*f*_cp_^*^ and Δ*f*_ca_ =  ± 0.5*f*_ca_^*^ for MBBA). *V*_c_(*ϕ*_N_) demonstrates a minimal peak at *ϕ*_N_^*^ that provides the maximal difference between *V*_0_ and *V*_c_ (i.e., *α* = *V*_0_ − *V*_c_) at *ϕ*_N_^*^. Thus, the maximal performance of the EC pattern is obtained at *ϕ*_N_^*^, resulting from a maximal angle *φ* (Fig. 1a). Accordingly, a rest state (*φ* = 0) held at *ϕ*_N_ = 0 (i.e., *α* < 0) changes into EC (i.e., *α* > 0) when *ϕ*_N_ increases, which then disappears at high *ϕ*_N_ (i.e., *α* < 0). Therefore, the performance (*φ*) of the EC patterns showed a typical *bell-shaped* type for SR^25^. Conversely, as shown in Fig. 1d, a colored amplitude noise *V*_N_ (*f*_ca_ ~ *f*_ca_^*^) provides a reversed nonmonotonic function *V*_c_(*V*_N_) that indicates ISR^17^. Thus, EC (*φ* ≠ 0) generated at *V*_N_ = 0 (i.e., *α* > 0) disappears (i.e., *φ* = 0) around *V*_N_ = *V*_N_^*^ (i.e., *α* < 0) and then reappears (i.e., *φ* ≠ 0) at *V*_N_ >> *V*_N_^*^ (i.e., *α* > 0). In contrast to noise with *f*_cp_ ~ *f*_cp_^*^ (and *f*_ca_ ~ *f*_ca_^*^), noise with *f*_cp_ > *f*_cp_^*^ (and *f*_ca_ > *f*_ca_^*^) induces a monotonic increase in *V*_c_(*ϕ*_N_) [and *V*_c_(*V*_N_)], whereas noise with *f*_cp_ < *f*_cp_^*^(and *f*_ca_ < *f*_ca_^*^) induces a monotonic decrease in *V*_c_(*ϕ*_N_) [and *V*_c_(*V*_N_)], as shown in Figs. 1c and 1d, respectively. In these cases, SR and ISR cannot be obtained. For extremely high *f*_c_ [i.e., white noise with *f*_cp_ → ∞ (and *f*_ca_ → ∞)], the occurrence of EC is suppressed by the completely random action of noises on the motion of *q* and *ψ* in Eqs. (2) and (3)^37,39^. Note that although SR and ISR can be found independently, they do not transit between each other.

## Results

### Numerical results for the EC threshold

**Figure 2:**
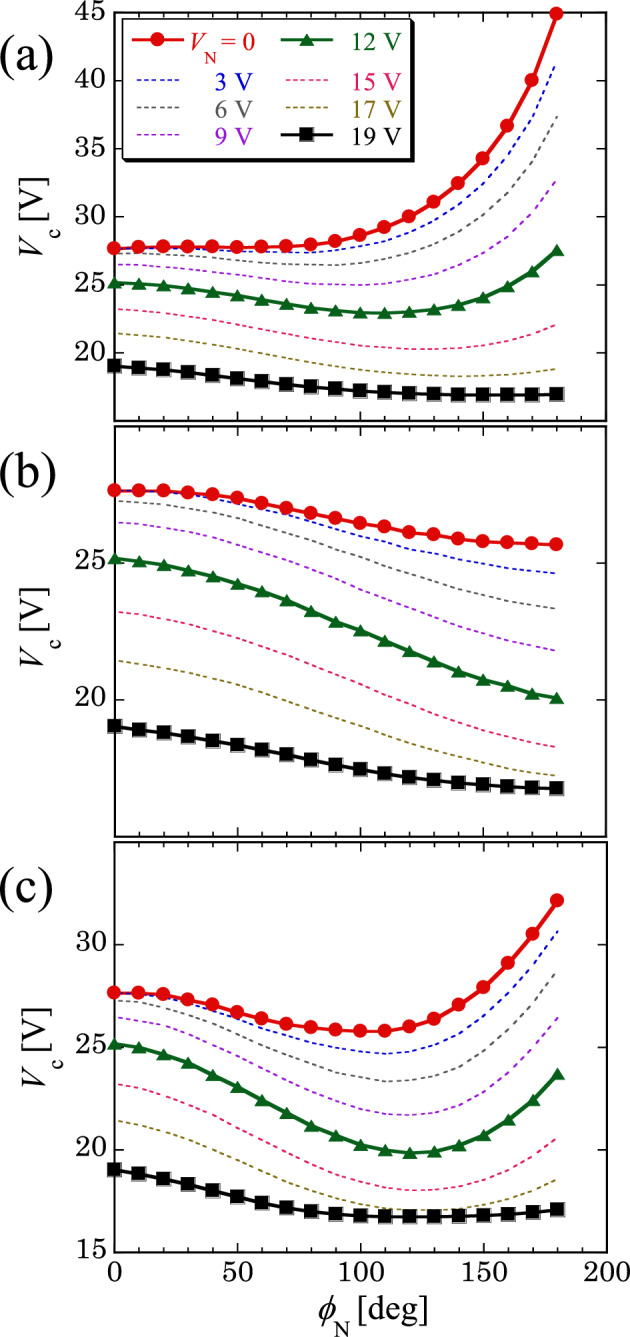
Behavior of *V*_c_(*ϕ*_N_) for various amplitude-noise intensities *V*_N_ with *f*_ca_ < *f*_ca_^*^. In the case of *f*_0_ = 2.5 kHz and *f*_ca_ = 100 Hz (< *f*_ca_^*^ ≈ 1 kHz), *V*_c_ was determined using the phase noise with (**a**) *f*_cp_ = 1 kHz (> *f*_cp_^*^ ≈ 100 Hz), (**b**) *f*_cp_ = 50 Hz (< *f*_cp_^*^), and (**c**) *f*_cp_ = 100 Hz (≈ *f*_cp_^*^). A nonmonotonic *V*_c_(*ϕ*_N_) [e.g., at *V*_N_ = 12 V in (**a,c**)] indicates SR, as shown in Fig. 1c. When *V*_N_ is increased, a monotonic increase in *V*_c_(*ϕ*_N_) in (**a**) smoothly changes into a monotonic decrease through a nonmonotonic behavior. In contrast, the nonmonotonic behavior of *V*_c_(*ϕ*_N_) in (**c**) changes into a monotonic decrease for high *V*_N_ (≥ 19 V).

The EC threshold (*V*_c_) was examined in the presence of both noises. Figure 2 demonstrates the phase noise-induced behavior of *V*_c_(*ϕ*_N_) for different amplitude noise intensities *V*_N_. Considering that the three cases of *V*_c_(*ϕ*_N_,*V*_N_ = 0) depend on *f*_cp_ (Fig. 1c), we examined *V*_c_(*ϕ*_N_) at *V*_N_ > 0 for *f*_cp_ > *f*_cp_^*^, *f*_cp_ < *f*_cp_^*^, and *f*_cp_ ~ *f*_cp_^*^, as shown in Fig. 2a–c, respectively. We also considered *V*_c_(*ϕ*_N_) at *V*_N_ = 0 for each case as a reference. As shown in Fig. 2a, *V*_c_(*ϕ*_N_), which shows a monotonic increase at *V*_N_ = 0, dramatically changes with increasing *V*_N_ and provides a nonmonotonic *V*_c_(*ϕ*_N_) for *V*_N_ ≥ 6 V. However, for higher values of *V*_N_ (> 17 V), *V*_c_(*ϕ*_N_) exhibits a monotonic decrease. This result indicates that *V*_N_ can change the phase noise-induced monotonic increase in *V*_c_(*ϕ*_N_) into nonmonotonic behavior, indicating SR. Consequently, *V*_N_ can control *V*_c_(*ϕ*_N_) by using an appropriate combination of noises. In contrast, no remarkable change in *V*_c_(*ϕ*_N_) was observed at *f*_cp_ < *f*_cp_^*^ (Fig. 2b). Moreover, in the case of *f*_cp_ ~ *f*_cp_^*^ (Fig. 2c), the nonmonotonic *V*_c_(*ϕ*_N_) was maintained up to an appropriate intensity *V*_N_ but disappeared at higher *V*_N_ values (≥ 19 V). In Fig. 2, we used a colored amplitude noise with *f*_ca_ < *f*_ca_^*^ to obtain the abovementioned results. In addition, a different behavior of *V*_c_(*ϕ*_N_) was obtained when a colored amplitude noise with *f*_ca_ > *f*_ca_^*^ or *f*_ca_ ~ *f*_ca_^*^ was used (see below).

**Figure 3:**
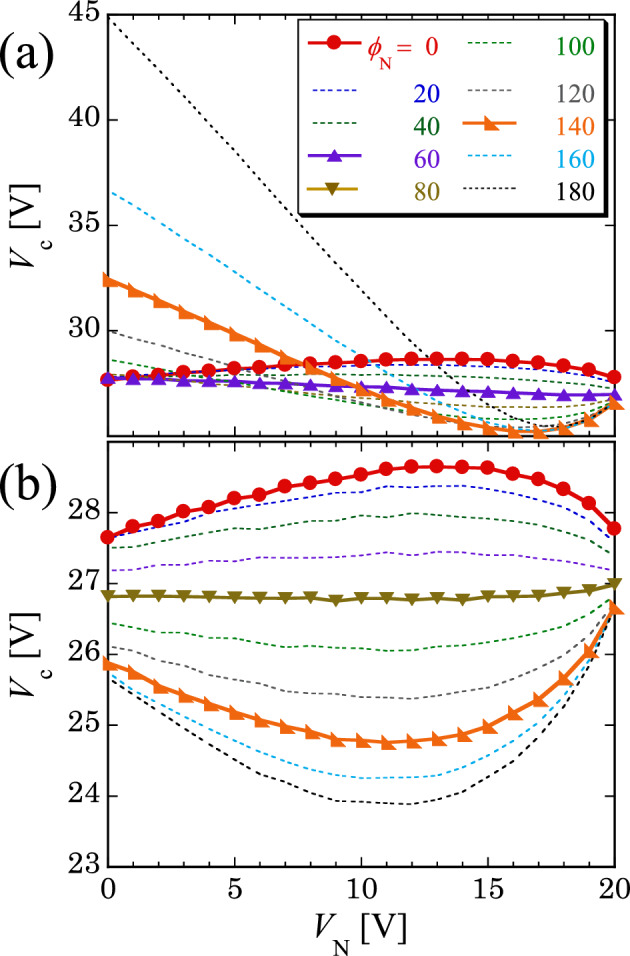
Behavior of *V*_c_(*V*_N_) for various phase-noise intensities *ϕ*_N_. In the case of *f*_0_ = 2.5 kHz and *f*_ca_ = 1 kHz (≈ *f*_ca_^*^≈ 1 kHz), *V*_c_ was determined using the phase noise with (**a**) *f*_cp_ = 100 Hz (≈ *f*_cp_^*^) and (**b**) *f*_cp_ = 50 Hz (< *f*_cp_^*^). The nonmonotonic *V*_c_(*V*_N_) [e.g., at *ϕ*_N_ = 140 deg in (**a,b**)] indicates SR. Furthermore, the reversed nonmonotonic *V*_c_(*V*_N_) [e.g., at *ϕ*_N_ = 0 in (**a,b**)] indicates ISR, as shown in Fig. 1d. With an increase in the *ϕ*_N_, the nonmonotonic behavior of *V*_c_(*V*_N_) smoothly changes to reversed nonmonotonic behavior by exhibiting a nearly constant behavior [at *ϕ*_N_ = 60 deg in (**a**) and *ϕ*_N_ = 80 deg in (**b**)]; thus, a transition from ISR to SR is found by increasing *ϕ*_N_.

The amplitude noise-induced behavior of *V*_c_(*V*_N_) for different phase intensities *ϕ*_N_ is shown in Fig. 3. This figure demonstrates more dramatic changes in *V*_c_(*V*_N_), which indicate a transition between SR and ISR. In the case of *f*_ca_ ~ *f*_ca_^*^ and *f*_cp_ ~ *f*_cp_^*^ (Fig. 3a), an increase in *ϕ*_N_ induced smooth changes to the nonmonotonic *V*_c_(*V*_N_), which indicates ISR for small *ϕ*_N_ values, to an almost constant *V*_c_(*V*_N_) and then to a reversed nonmonotonic *V*_c_(*V*_N_), indicating SR. Moreover, in the case of *f*_ca_ ~ *f*_ca_^*^ and *f*_cp_ < *f*_cp_^*^ (Fig. 3b), a similar change was observed; however, the detail of *V*_c_(*V*_N_) was different in each case. For example, an intersection of *V*_c_(*V*_N_) was found for *f*_cp_ ~ *f*_cp_^*^(Fig. 3a), indicating that a peculiar value of *V*_c_ could be determined by two ways using different *ϕ*_N_ (at a fixed *V*_N_).

**Figure 4:**
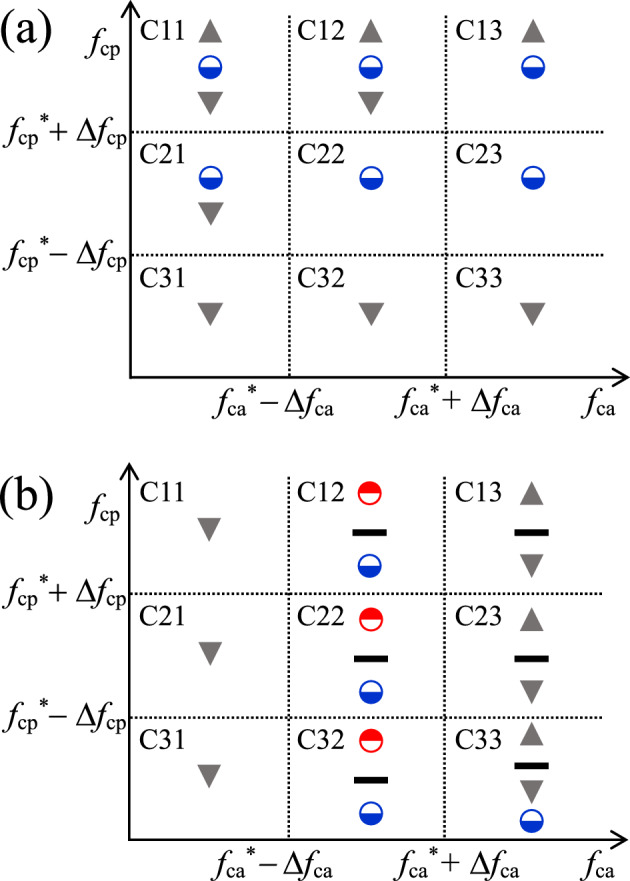
Phase diagrams of *V*_c_ in the *f*_ca_ and *f*_cp_ planes. (**a**) *V*_c_(*ϕ*_N_) for various *V*_N_ values. Notably, the symbols indicating *V*_c_(*ϕ*_N_) are arranged in the order in which *V*_N_ increases from top to bottom. Case11 (C11), C21 and C31 correspond to Fig. 2a, c, b, respectively. (**b**) *V*_c_(*V*_N_) for various *ϕ*_N_ values. The symbols indicating *V*_c_(*V*_N_) are arranged in the order in which *ϕ*_Ν_ increases from top to bottom. C12 and C32 correspond to Fig. 3a, b, respectively. The symbols filled triangle, downward filled triangle, and filled rectangle indicate monotonic increase, monotonic decrease, and constant behavior of *V*_c_, respectively; blue circle filled at bottom and red circle filled at top indicate nonmonotonic and reversed nonmonotonic behavior of *V*_c_, providing SR and ISR, respectively. In this numerical study, *f*_0_ = 2.5 kHz,* f*_ca_^*^ ≈ 1 kHz, *f*_cp_^*^ ≈ 100 Hz, Δ*f*_ca_ ≈ 500 Hz, and Δ*f*_cp_ ≈ 50 Hz. See Supplementary Information for details.

Finally, *V*_c_(*ϕ*_N_) and *V*_c_(*V*_N_) in the *f*_ca_ and *f*_cp_ plane are shown in Fig. 4a, b, respectively. The case11 (C11) presented in Fig. 4a indicates the *V*_c_(*ϕ*_N_) shown in Fig. 2a, whereas C32 presented in Fig. 4b indicates the *V*_c_(*V*_N_) shown in Fig. 3b. For *V*_c_(*ϕ*_N_), SR appears for wide regions of *f*_cp_ and *f*_ca_ by controlling *V*_N_, except for *f*_cp_ < *f*_cp_^*^ − Δ*f*_cp_. For *V*_c_(*V*_N_), SR and ISR appear concurrently, and their transitions are indicated by the constant behavior symbol [although limited to narrow regions of *f*_ca_ (i.e., *f*_ca_^*^ − Δ*f*_ca_ < *f*_ca_ < *f*_ca_^*^ + Δ*f*_ca_)]. In addition, an almost constant behavior of *V*_c_(*V*_N_) was found during the transition between SR and ISR for *f*_ca_^*^ − Δ*f*_ca_ < *f*_ca_ < *f*_ca_^*^ + Δ*f*_ca_ and during the change between monotonic increase and decrease for *f*_ca_ > *f*_ca_^*^ + Δ*f*_ca_.

### Experimental results for SR and ISR

**Figure 5:**
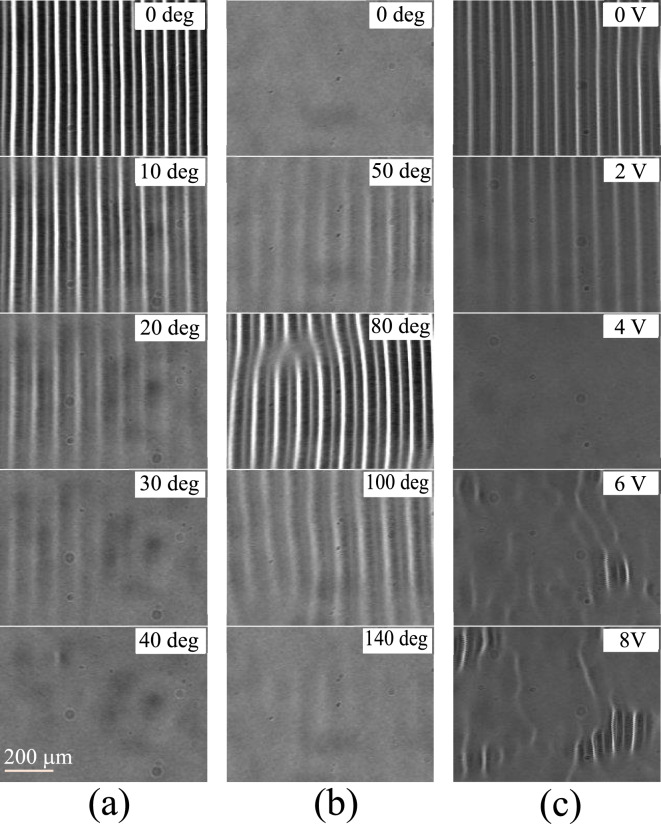
EC pattern evolutions with increasing *ϕ*_N_ or *V*_N_. ECs were observed at *f*_0_ = 1.5 kHz in a cell (MBBA, *d* = 25 μm). Phase noise *ϕ*_N_ with *f*_cp_ = 2 kHz smoothly increased when (**a**) *V*_0_ = 18.8 V and *V*_N_ = 0 and (**b**) *V*_0_ = 16.5 V and *V*_N_ = 5 V with *f*_ca_ = 1 kHz. (**c**) Amplitude noise *V*_N_ with *f*_ca_ = 4.5 kHz smoothly increased when *V*_0_ = 18.3 V and *ϕ*_N_ = 90 deg with* f*_cp_ = 1.6 kHz. Notably, (**a,b**) show pattern evolutions for *V*_c_(*ϕ*_N_) at *V*_N_ = 0 and* V*_N_ = 12 V in Fig. 2a, respectively, and (**c**) indicates that for *V*_c_(*V*_N_) at *ϕ*_N_ = 20 deg in Fig. 3b. SR and ISR are observed in (**b,c**), respectively. See Supplementary Information for details.

In an electro-optical system for EC^35,36^, we observed the emergence of SR and ISR by controlling both noises (i.e., *ϕ*_N_ and *V*_N_) with appropriate *f*_cp_ and *f*_ca_ values. For reference, a conventional pattern evolution with increasing *ϕ*_N_ (at *V*_N_ = 0) was found at *V*_0_ = 18.8 V [> *V*_c_(*V*_N_ = 0) = 17.8 V], as shown in Fig. 5a [see the corresponding *V*_c_(*ϕ*_N_, *V*_N_ = 0) in Fig. 2a]. Since *α* decreased with increasing *ϕ*_N_, the performance of EC patterns (or optical intensity *I* ∝ *φ*^2^)^40^ decreased with increasing *ϕ*_N_. Then, EC disappeared at *ϕ*_N_ = 40° for *α* < 0 (i.e., *V*_0_ < *V*_c_). Such a pattern evolution is trivial and intuitive in the presence of conventional noise. Conversely, at *V*_0_ = 16.5 V [< *V*_c_(*V*_N_ = 5 V) = 17.1 V], SR was found, as shown in Fig. 5b [see the corresponding *V*_c_(*ϕ*_N_, *V*_N_ = 12 V) in Fig. 2a]. By increasing *ϕ*_N_, *α* < 0 changed to *α* > 0 and then again to *α* < 0. Thus, EC smoothly appeared and disappeared with increasing *ϕ*_N_. The EC performance (i.e., *φ*) showed a typical *bell-shaped* curve (i.e., SR), which is similar to the SR obtained from a single phase noise^25^.

Figure 5c shows a unique pattern evolution with increasing *V*_N_ at *ϕ*_N_ = 90° and *V*_0_ = 18.3 V [> *V*_c_ (*V*_N_ = 0) = 17.8 V]. A typical *reversed bell-shaped* curve of the EC performance indicating ISR was observed, which is similar to that of the ISR obtained from a single amplitude noise^17^. In addition, the present patterns at high *V*_N_ (= 6 − 8 V) showed localized ECs, which were not observed in a previous study based on a single amplitude noise^17^. Such localized patterns are attributed to the combined effect of both noises, indicating that comparatively high intensities of both noises may play a role in EC pattern structures as well as EC thresholds. In localized ECs that are stationary (not transient), a noise-induced abnormal distribution of electric charges for ECs may occur^41^, which can be distinguished from the normal distribution of conventional ECs^34^. Such localized ECs are beyond the scope of our analysis using Eqs. (2) and (3). Evidently, our experimental observations revealed the crucial effects of colored amplitude and phase noises on the generation of both resonances through the smooth variation of the EC threshold. Unfortunately, the transition between SR and ISR (Fig. 3) was not observed due to experimental limitations, such as material parameters [e.g., *τ*, *σ*_H_, λ, and *η* in Eqs. (2) and (3)] that are highly sensitive to *V*_c_ but difficult to tune to the values in the numerical study.

## Discussion and conclusion

During the last four decades, SR has been intensively investigated in useful concepts of randomness^42^ and extensively applied for noise benefits^42–44^. In this study, we presented an SR transitioned from ISR which has been less addressed so far in the literature. By examining the threshold of ac-driven EC, we showed that a suitable combination of colored amplitude and phase noises could induce the emergence of both resonances and the transition between them. Therefore, we demonstrated that SR and ISR, which have been independently reported so far, could be handled in a single framework. A recent numerical study on the co-occurrence of SR and ISR^16^ reported in a neural system should be distinguished from this study. Such co-occurrence implies that an ISR exhibiting the minimal output for one performance measure (mean firing rate) can induce an SR exhibiting the maximal output for another performance measure (mutual information). Note that colored noise can vary the probability density of the state of systems, which is governed by the Fokker–Planck equation^45^. According to the correlation time of colored noise, the probability density distribution can also provide two peaks for two possible states (*φ* = 0 and *φ* ≠ 0 in this study)^45^.

In the Carr–Helfrich mechanism of ac-driven EC^32–36^, the combined electric noises can play critical roles in the occurrence of EC through their effects on the motion of electric charges (by Coulomb force against the electro-elastic restoring force of the NLC) and vary the EC threshold. In particular, in the appropriate conditions of correlation times and intensities of the two noises, their roles can contradict each other, i.e., one may suppress EC and the other may promote EC. Consequently, this competition between the noise-induced stabilization and destabilization effects on EC is the underlying reason for the emergence of both resonances and their transition. If there exists a nonequilibrium, free energy-like potential^46^ with its minima at *φ* = 0 and *φ* ≠ 0 (i.e., a rest state and a convection state, respectively), which correspond to an ice state and a warm state, respectively, in the study of ice-age cycles^2,3^, such a potential should be investigated along with its symmetry breaking^1,19^ to understand the detail of the competition mechanism; and this is an important open question. Our numerical and experimental findings suggest that the control of SR and ISR by combining amplitude and phase noises can be very useful for electrical applications, such as sensing technologies^36,47,48^ and brain science^12,13^, for which additional phase noise can be readily introduced. Furthermore, the transition between SR and ISR may provide effective controls for desired and undesired performances in various related fields^49,50^.

### Supplementary Information

Supplementary Information.

## Data Availability

The data that support the findings of this study are available from the corresponding author upon reasonable request.

## References

[1] Zhang X-J, Qian H, Qian M (2012). Stochastic theory of nonequilibrium steady states and its applications. Part I. Phys. Rep..

[2] Benzi R, Sutera A, Vulpiani A (1981). The mechanism of stochastic resonance. J. Phys. A.

[3] Benzi R, Parisi G, Sutera A, Vulpiani A (1982). Stochastic resonance in climate change. Tellus.

[4] Dodda A (2020). Stochastic resonance in MoS_2_ photodetector. Nat. Commun..

[5] Bhar B, Khanna A, Parihar A, Datta S, Raychowdhury A (2020). Stochastic resonance in insulator–metal–transition systems. Sci. Rep..

[6] Hohmann W, Müller J, Schneider FW (1996). Stochastic resonance in chemistry. J. Phys. Chem..

[7] Suzuki Y, Asakawa N (2022). Stochastic resonance in organic electronic devices. Polymers.

[8] Douglass JK, Wilkens L, Pantazelou E, Moss F (1993). Noise enhancement of information transfer in crayfish mechanoreceptors by stochastic resonance. Nature.

[9] Russell DF, Wilkens LA, Moss F (1999). Use of behavioral stochastic resonance by paddle fish for feeding. Nature.

[10] Simonotto E, Riani M, Seife C, Roberts M, Twitty J, Moss F (1997). Visual perception of stochastic resonance. Phys. Rev. Lett..

[11] Roy PK, Rallabandi VPS (2010). Magnetic resonance imaging (MRI) enhancement using stochastic resonance. Magn. Reson. Imaging.

[12] Gluckman BJ, Netoff TI, Neel EJ, Ditto WL, Spano ML, Schiff SJ (1996). Stochastic resonance in a neuronal network from the mammalian brain. Phys. Rev. Lett..

[13] Kai S, Mori T (2002). Noise-induced entrainment and stochastic resonance in human brain waves. Phys. Rev. Lett..

[14] Gutkin B, Jost J, Tuckwell HC (2009). Inhibition of rhythmic neural spiking by noise: the occurrence of a minimum in activity with increasing noise. Naturwissenschaften.

[15] Uzuntarla M, Cressman JR, Ozer M, Barreto E (2013). Dynamical structure underlying inverse stochastic resonance and its implications. Phys. Rev. E.

[16] Zamani A, Novikov N, Gutkin B (2020). Concomitance of inverse stochastic resonance and stochastic resonance in a minimal bistable spiking neural circuit. Commun. Nonlinear Sci. Numer. Simulat..

[17] Huh J-H (2016). Inverse stochastic resonance in electroconvection by multiplicative colored noise. Phys. Rev. E.

[18] Touboul JD, Staver AC, Levin SA (2018). On the complex dynamics of savanna landscapes. Proc. Natl. Acad. Sci. USA.

[19] Torres JJ, Uzuntarla M, Marro J (2020). A theoretical description of inverse stochastic resonance in nature. Commun. Nonlinear Sci..

[20] Sutera A (1981). On stochastic perturbation and long-term climate behavior. Q. J. R. Meteorol. Soc..

[21] Nicolis G, Altares V (1987). A new method of analysis of the effect of weak colored noise in nonlinear dynamical systems. J. Stat. Phys..

[22] Jia Y, Zheng X, Hu X, Li J (2001). Effects of colored noise on stochastic resonance in a bistable system subject to multiplicative and additive noise. Phys. Rev. E.

[23] Gammaitoni L, Marchesoni F, Menichella-Saetta E, Santucci S (1994). Multiplicative stochastic resonance. Phys. Rev. E.

[24] Seki K, Barzykin AV (1997). Stochastic resonance driven by Gaussian multiplicative noise. Europhys. Lett..

[25] Huh J-H, Yano Y, Miyagawa N (2019). Phase noise can induce stochastic resonance?. J. Phys. Soc. Jpn..

[26] Chowdhury A, Barbay S, Clerc MG, Robert-Philip I, Braive R (2017). Phase stochastic resonance in a forced nanoelectromechanical membrane. Phys. Rev. Lett..

[27] Huh J-H (2011). Noise-induced threshold shift and pattern formation in electroconvection by controlling characteristic time scales. Phys. Rev. E.

[28] Huh J-H, Kai S (2014). Colored noise-induced threshold shifts and phase diagrams in electroconvections. J. Phys. Soc. Jpn..

[29] Y. Yamamoto and D. Nozaki, Enhancement of stochastic resonance in a FitzHugh-Nagumo neuronal model driven by colored noise, Phys. Lett., A **243**, 281(1998).

[30] Nozaki D, Mar DJ, Grigg P, Collins JJ (1999). Effects of colored noise on stochastic resonance in sensory neurons. Phys. Rev. Lett..

[31] Guo D (2011). Inhibition of rhythmic spiking by colored noise in neural systems. Cogn. Neurodyn..

[32] Carr EF (1969). Influence of electric fields on the molecular alignment in the liquid crystal* p*-(anisalamino)-phenyl acetate. Mol. Cryst. Liq. Cryst..

[33] Helfrich W (1969). Conduction-induced alignment of nematic liquid crystals: Basic model and stability considerations. J. Chem. Phys..

[34] Smith IW, Galerne Y, Lagerwall ST, Dubois-Violette E, Durand G (1975). Dynamics of electrohydrodynamic instabilities in nematic liquid crystals. J. Phys (Paris).

[35] Prost J, de Gennes PG (1993). The Physics of Liquid Crystals.

[36] Eber N, Salamon P, Buka A (2016). Electrically induced patterns in nematics and how to avoid them. Liq. Cryst. Rev..

[37] Kawakubo T, Yanagita A, Kabashima S (1981). External noise effect on the onset of Williams domain in nematic liquid crystals. J. Phys. Soc. Jpn..

[38] Huh J-H (2015). Multiplicative noise effects on electroconvection in controlling additive noise by a magnetic field. Phys. Rev. E.

[39] Kai S, Kai T, Takata M, Hirakawa K (1979). Effect of the white noise on electrohydrodynamic transitions in nematics. J. Phys. Soc. Jpn..

[40] John T, Stannarius R (2004). Preparation of subharmonic patterns in nematic electroconvection. Phys. Rev. E.

[41] Huh J-H (2017). Traveling waves and worms in ac-driven electroconvection under external multiplicative noise. Phys. Rev. E.

[42] McDonnell MD, Abbott D (2009). What is stochastic resonance? Definitions, misconceptions, debates, and its relevance to biology. PLoS Comput. Biol..

[43] Gammaitoni L, Hänggi P, Jung P, Marchesoni F (1998). Stochastic resonance. Rev. Mod. Phys..

[44] Budrikis Z (2021). Forty years of stochastic resonance. Nat. Rev. Phys..

[45] Sancho, J. M. & Miguel, M. S. Langevin equations with colored noise. In *Noise in Nonlinear Dynamics Systems*. Vol. 1. *Theory of Continuous Fokker−Planck Systems *(Eds. Moss, F., McClintock, P. V. E.) (Cambridge University Press, 1989) (Lugiato, L. A., Broggi, G., Merri, M. & Pernigo, M. A. Control of noise and applications to optical systems. Ibid. Vol. 2. *Theory of Noise-Induced Processes in Special Applications*).

[46] Wio HS, Deza JI, Sanchez AD, Garcia-Garcia R, Gallego R, Revelli JA, Deza RR (2022). The nonequilibrium potential today: A short review. Chaos Solit. Fract..

[47] Collins JJ, Imhoff TT, Grigg P (1997). Noise-mediated enhancements and decrements in human tactile sensation. Phys. Rev. E.

[48] Dong H, He K, Shen X, Ma S, Wang H, Qiao C (2020). Adaptive intrawell matched stochastic resonance with a potential constraint-aided line enhancer for passive sonars. Sensors.

[49] Horsthemke W, Lefever R (1984). Noise-Induced Transitions.

[50] Ridolfi L, D'Odorico P, Laio F (2011). Noise-Induced Phenomena in Environmental Sciences.

