# Supplementary material for: Control of stochastic and inverse stochastic resonances in a liquid-crystal electroconvection system using amplitude and phase noises
Source: Sci Rep. 2023 Oct 6;13:16883. doi: 10.1038/s41598-023-44043-4 (PMC10558573; doi:10.1038/s41598-023-44043-4)
Supplement: Supplementary file 1 — Supplementary Information. [file 41598_2023_44043_MOESM1_ESM.pdf]

Scientific Reports Supplementary Information

Control of stochastic and inverse stochastic resonances in a liquid-crystal electroconvection system using amplitude and phase noises

Jong-Hoon Huh*, **Masato Shiomi, and Naoto Miyagawa**

Department of Physics and Information Technology, Faculty of Computer Science and Systems Engineering, Kyushu Institute of Technology, Fukuoka 820-8502, Japan

*Corresponding author: huh@phys.kyutech.ac.jp

Supplementary Methods

Numerical calculation. We used the discrete fourth-order Runge-Kutta method in a versatile software package (MATLAB R2020b) to determine the threshold voltage V_c of electroconvection (EC) from the governing Carr-Helfrich Eqs. (2) and (3). V_c can be determined as the lowest value V in the numerical loop with an increment of $\Delta V = 0.1$ V for which the director angle φ does not relax to zero [34]. The colored noise, with a cutoff frequency f_{ca} for amplitude noise or f_{cp} for phase noise, was provided by a built-in frequency filtering program within the software. The parameters for the nematic liquid crystal (NLC) [i.e., *p*-methoxybenzylidene-*p*'-*n*-butylaniline (MBBA)] and the control parameters are shown in Table 1 [34,35].

Table 1. Material and control parameters used in this study.

material parameters (SI units) *1)			control parameters		
	calculation	experiment		calculation	experiment
$\varepsilon_{//}$	4.605		f_0	2.5 kHz	1.5 kHz
$\varepsilon_{\perp}$	5.032	5.16	V_0	10–50 V	10–20V
$\sigma_{//}$	6.14×10^{-8}		f_{cp}	0.05–5 kHz	1.6 kHz
$\sigma_{\perp}$	5.13×10^{-8}	1.13×10^{-8}	f_{ca}	0.05–2 kHz	4.5 kHz
γ_1	0.0755				
γ_2	−0.0785		ϕ_N	0–180 deg	0–180 deg
α_v	0.1986				
K_{33}	6.53×10^{-12}		V_N	0–20 V	0–10 V
k	1.26×10^{5}				

*1) see Refs. 34 and 35 for the details of parameters for Eqs. (2) and (3): conductivity anisotropy $\sigma_a = \sigma_{//} - \sigma_{\perp}$, dielectric anisotropy $\varepsilon_a = \varepsilon_{//} - \varepsilon_{\perp}$, charge relaxation time $\tau = (\varepsilon_0\varepsilon_{//})/\sigma_{//}$, director relaxation constant λ, Helfrich parameter σ_H, equivalent field E_0 including elastic constant K_{33} and wavenumber k of EC, and effective viscosity η determined by real viscosity constants γ_1, γ_2 and α_v: $\sigma_H = \sigma_{//}\left(\frac{\varepsilon_{\perp}}{\varepsilon_{//}} - \frac{\sigma_{\perp}}{\sigma_{//}}\right)$, $\lambda = \varepsilon_0 |\varepsilon_a| \frac{\varepsilon_{\perp}}{\varepsilon_{//}}\left(\frac{1}{\gamma_1} + \frac{1}{\eta_0}\right)$, $E_0^2 = \frac{\varepsilon_{//}}{\varepsilon_0|\varepsilon_a|\varepsilon_{\perp}} K_{33}k^2$,

$\frac{1}{\eta} = \frac{1}{\eta_0}\left(\frac{2\gamma_1}{\gamma_1 - \gamma_2}\right) + \frac{|\varepsilon_a|}{\varepsilon_{//}}\left(\frac{1}{\gamma_1} + \frac{1}{\eta_0}\right)$, $\eta_0 = \left(\frac{\gamma_1}{\alpha_2}\right)^2\left(\frac{1}{2}\alpha_v - \frac{\alpha_2^2}{\gamma_1}\right)$, $\alpha_2 = \frac{1}{2}(\gamma_2 - \gamma_1)$.

In general, the threshold voltage V_c can be calculated in the pure ac field with a frequency f_0 by the following equation:

$$V_c^2(f_0) = V_0^2(1 + 4\pi^2 f_0^2\tau^2)/[\delta^2 - (1 + 4\pi^2 f_0^2\tau^2)].$$

Here, τ and δ^2 correspond to the charge relaxation time and the Helfrich parameter, respectively [34,35]. In the presence of noise [Eq. (4)], the variation of V_c was numerically determined as a function of the amplitude noise intensity V_N and the phase noise intensity ϕ_N.

Experimental procedure. A typical NLC (i.e., MBBA) [34,35] was injected into a planar alignment cell with two parallel transparent indium tin oxide electrodes (E.H.C., Ltd, Japan). In this cell, the director $\boldsymbol{n}$ ($|\boldsymbol{n}| = 1$, $\boldsymbol{n} \equiv -\boldsymbol{n}$), which indicates a locally averaged direction of rod-like molecules in the NLC, homogenously tends to the preferred direction (the $\boldsymbol{x}$ axis in this study) before EC (i.e., for $V < V_c$). The application of an initial voltage $V_0(t)[= E(t)d = \sqrt{2}V_0 \cos 2\pi f_0 t]$ across the thin NLC layer with a thickness d (d = 25 μm in this study) [$\boldsymbol{E}(t)$ // $\hat{z}$] electrohydrodynamically destabilizes the anisotropic fluid (i.e., NLC) by the Carr-Helfrich mechanism [32-36]. The EC was observed at V_c by the lens effect of the periodic director modulation [35,36]. The function $V_c(f_0)$ was experimentally confirmed in one- [51,52] and two- [35,36,53] dimensional cells. To generate colored amplitude and phase noises, two noise generators (HSA4051, NF) were used, which could control the cutoff frequencies f_{ca} and f_{cp} through low pass filters [54,55]. The filters allow low-frequency components to pass through but attenuate components with frequencies higher than f_{ca} and f_{cp}. The noise generated by NG-2 was introduced into the original wave generator SWG (WF1974, NF), which played a role as phase noise; the amplitude noise was generated by NG-1and combined through a combiner (50PD-016, JFW) with the voltage modulated by phase noise, as shown in Fig. 1e. The optical patterns for EC were observed in the *xy* plane using computer-controlled image software (Scion Image) and an image-capture board (PCI-VE5, Scion Corp.) together with a charge-coupled device camera (XC-75, Sony) mounted on a polarizing microscope (ML9300, Olympus). The experiment was carried out at a stable temperature (T = 25 ± 0.2 °C) using a temperature controller (TH-99, Japan Hightech).

References

51. J. -H. Huh and S. Kai, Electroconvection in nematic liquid crystals in Hele-Shaw cells, Phys. Rev. E **68**, 042702 (2003).
52. J. -H. Huh, Electroconvection in one-dimensional liquid crystal cells, Phys. Rev. E **97**, 042707 (2018).
53. K. Hirakawa and S. Kai, Analogy between hydrodynamic instabilities in nematic liquid crystal and classical fluid, Mol. Cryst. Liq. Cryst. **40**, 261 (1977).
54. J. -H. Huh, Influence of external noise on various electrohydrodynamic instabilities in a nematic liquid crystal, J. Phys. Soc. Jpn. **81**, 104602 (2012).
55. O. J. Ushie, M. Abbod, E. C. Ashigwuike, and S. Lawan, Constrained nonlinear optimization of unity gain operational amplifier filters using PSO, GA and Nelder-Mead, Inter. J. Intelligent Control and Systems, **20**, 26 (2015).

Supplementary Results

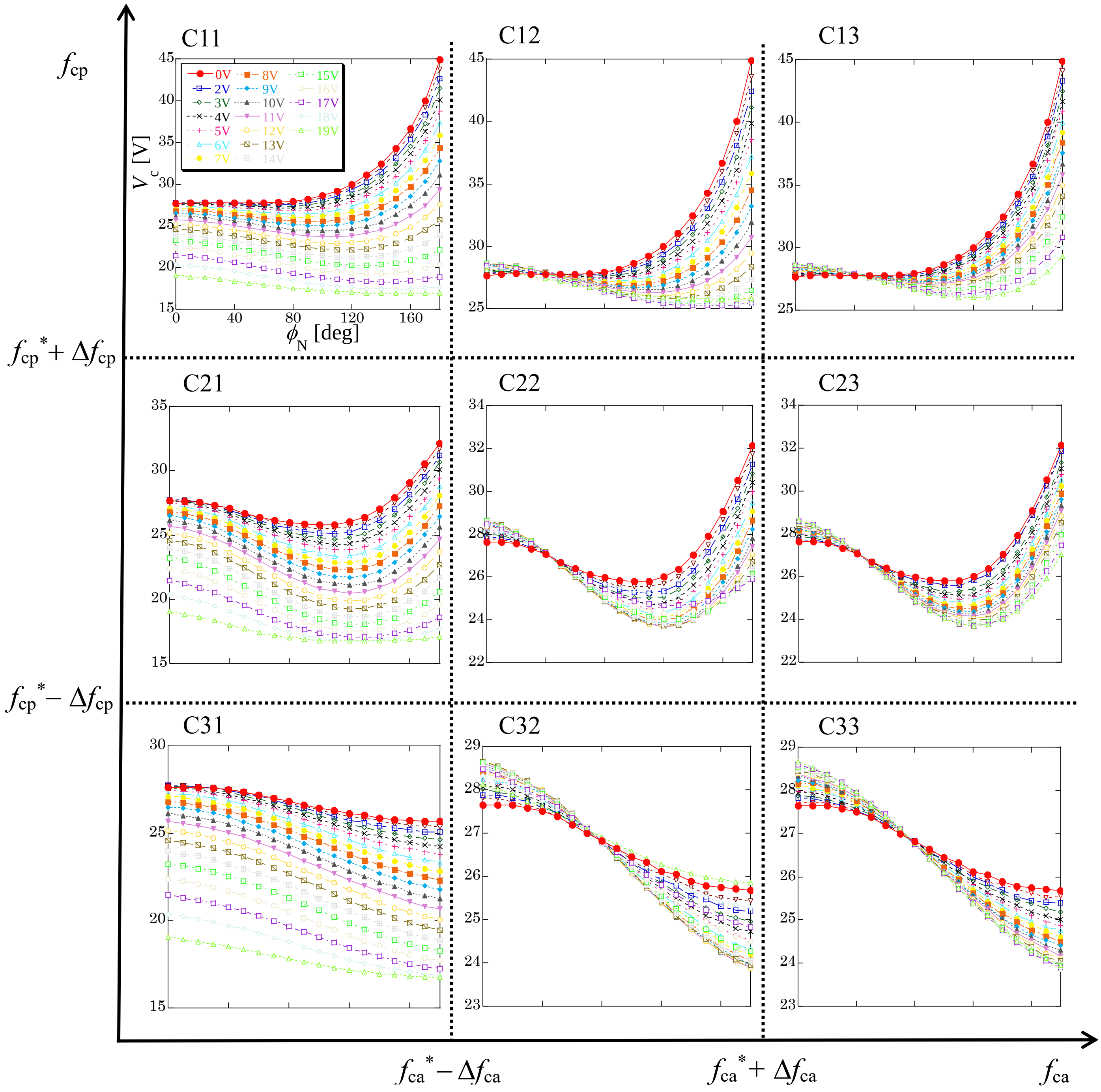

Supplementary FIG. (a) The threshold function $V_c(\phi_N)$ for various V_N values in the f_{ca} and f_{cp} plane. This corresponds to Fig. 4a.

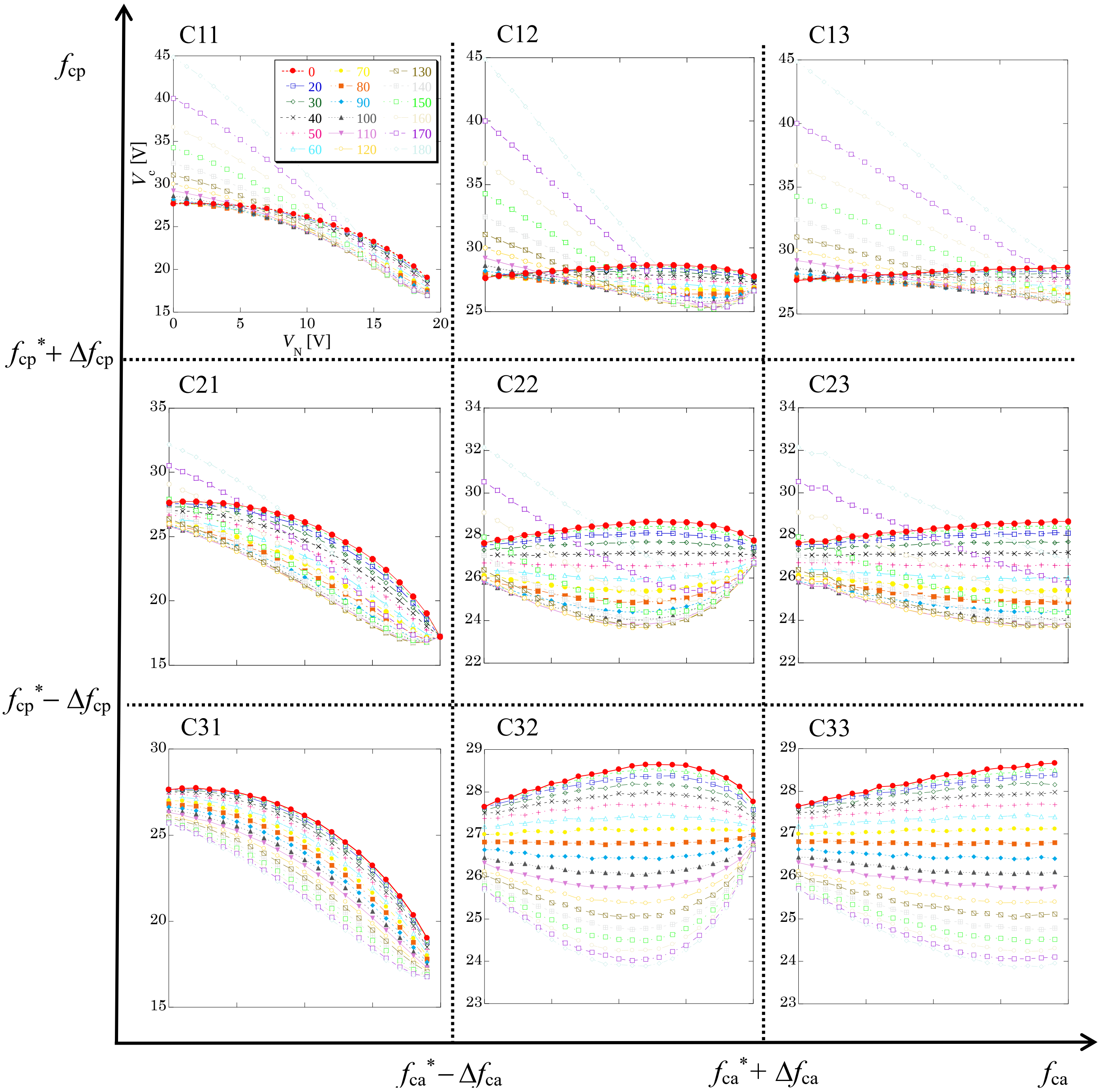

Supplementary FIG. (b) The threshold function $V_c(V_N)$ for various ϕ_N values in the f_{ca} and f_{cp} plane. This corresponds to Fig. 4b.
